# Increased interleukin-8 levels in cord blood serum of newborn—possible effect of cytokine dysregulation in pregnancies complicated by rheumatic disease on newborn vasculature

**DOI:** 10.3389/fimmu.2026.1931512

**Published:** 2026-09-08

**Authors:** Klara Rosta, Antonia Mazzucato-Puchner, Patrick Zommer, Christina Hörhager, Mira Horvath, Valerie Kuczwara, Valentin Ritschl, Daniel Zimpfer, Barbara Messner, Ulrike Baranyi

**Affiliations:** 1 Department of Obstetrics and Gynecology, Division Gynecological Endocrinology and Reproductive Medicine, Medical University of Vienna, Vienna, Austria; 2 Department of Internal Medicine III, Division of Rheumatology, Medical University of Vienna, Vienna, Austria; 3 Cardiac Surgery Research Laboratory, Department of Cardiac and Thoracic Aortic Surgery, Medical University of Vienna, Vienna, Austria; 4 Institute of Outcomes Research, Center of Medical Data Science, Medical University of Vienna, Vienna, Austria; 5 Clinical Department of Cardiac and Thoracic Aortic Surgery, Medical University of Vienna, Vienna, Austria

**Keywords:** cardiovascular disease, cytokines, inflammatory rheumatic disease, offspring, umbilical cord, vasculature, pregnancy

## Abstract

**Introduction:**

Systemic autoimmune rheumatic diseases (SARDs) are associated with a higher risk of cardiovascular diseases. During pregnancy, maternal immune disbalance might impact the vasculature of the newborn. We, therefore, aimed to investigate a possible association between maternal inflammatory rheumatic disease and inflammatory signaling in the umbilical cord of the newborn.

**Methods:**

This single-center study included neonates of women with connective tissue disorders (CTDs) and inflammatory joint diseases (IJDs) and healthy controls (HC).

**Results:**

The tissue of umbilical cords (UCs) of neonates born to IJD patients showed elevated ICAM-1 expression, indicating increased inflammatory response. Monocyte subsets in venous umbilical cord blood (VUCB) showed an equal distribution of non-classical monocytes in all groups. Leukocytes in VUCB showed no significant differences between the three groups although a trend of Treg reduction and an increase in cytotoxic T lymphocytes (CTLs) in the IJD offspring group were detectable. Cytokine levels of VUCB serum were overall low, except IL-8 with significantly elevated concentrations in the IJD neonate group. In the maternal serum of IJD patients, IL-8 levels were low comparable to IL-8 in healthy umbilical cord vein (UCV) serum, indicating a *de novo* synthesis of IL-8 in the UCV of neonates. Platelet-derived growth factor AA (PDGF-AA) was significantly reduced in the sera of IJD and CTD offspring. Furthermore, a significant reduction of human UCV smooth muscle cell (HUVSMC) proliferation incubated with plasma of the VUCB-IJD sera of neonates, possibly an effect of reduced PDGF-AA levels, is detectable.

**Conclusion:**

Well-controlled SARD during pregnancy does not seem to influence most of the inflammatory signaling in the UC. However, the elevated levels of IL-8 in the serum of neonates of women with IJD might have an impact on long-term vascular health and cardiovascular events.

## 1 Introduction

Systemic autoimmune rheumatic diseases (SARDs), such as inflammatory rheumatic diseases (IRDs), autoimmune connective tissue disorders (CTDs), and inflammatory joint diseases (IJDs), are accompanied with a higher risk of cardiovascular disease (CVD) (1, 2). The role of chronic inflammation on the vasculature and genetic predisposition has been suggested as possible causes (3–5). Autoimmune disorders often affect women in their reproductive age. There has been a paradigm change in the treatment of SARD during pregnancy, and thanks to the development of new biologics and data about medication use in pregnancy, more and more women with SARD can fulfil their family planning (6). In pregnancy, women with IJD like rheumatoid arthritis (RA) or CTD like systemic lupus erythematosus (SLE) or SS are treated with pregnancy-compatible immunomodulatory medications to avoid teratogenicity but reach remission or very low and stable disease activity during pregnancy in order to minimize risk of flare and complications in the course of the pregnancy (7, 8). Nevertheless, the pathognomical immunological signature of these diseases and the disbalance of immune cells and antibodies pose a theoretical risk on the fetus due to the transplacental transfer of autoantibodies, cytokines, extracellular vesicles, and their content through the feto-maternal interface, which might have a negative effect on the vasculature of the newborn. Inflammatory cytokines with vascular remodeling potential might have an influence on the development of pregnancy complications and also on the vascular function of the newborn. Elevated IL-8/CXCL8 concentration was detected *in vitro* in HUVEC cells of preeclamptic patients and has been associated with endothelial dysfunction, increased vascular permeability, and altered cell migration, all of which influence the development of pregnancy complications and vascular function (9). Differently, IL-8-producing T cells detected in the blood of preterm neonates suggested a role in early defense against infections (10). It is debated if cytokines cross the placenta or will be generated locally in the developing immune system of the newborn (11). Inflammation due to infection or autoimmune diseases seems to affect the cytokine levels of the newborn. Inflammatory cytokines are detectable in response to maternal infection even in the absence of pathogenic DNA in the fetal circulation, which suggests a possible transfer mechanism of TNF-α and other cytokines from the maternal side (7).

Active rheumatic disease in pregnancy leads to increased risk of miscarriages, preeclampsia, fetal growth restriction, and preterm birth in women with inflammatory joint diseases (12). Quiescent disease activity achieved through the new treatment modalities during pregnancy reduces the risk of these obstetric complications (13, 14).

The transfer of autoantibodies and possible increased levels of inflammatory factors seems to induce increased levels of congenital heart block in the offspring of SSA/SSB-positive patients (15). It is also suggested that long-term inflammatory alterations might induce structural changes in the developing vasculature and heart (16).

In this study, we analyzed the cytokine and immune cell profile in the umbilical cord blood from pregnancies with SARD compared to umbilical cord blood from healthy pregnancies. Additionally, we determined the inflammatory and oxidative state of vascular cells of the umbilical cord tissue. These data might provide insight to the effect of treated inflammation to the vasculature of the offspring, as well as to the pathomechanism of vascular dysfunction in pathologic pregnancies.

Our study gives insight to the early effects of autoimmune diseases on the offspring, especially on their vasculature and humoral and cellular immunocompetency.

## 2 Materials and methods

### 2.1 Study design

Pregnant patients suffering from inflammatory joint disease [RA, axial spondyloarthritis (axSpA), and psoriasis arthritis (PsoA)] and connective tissue disorders (SLE, SS, and systemic sclerosis) with a planned delivery at the Medical University of Vienna (MUV) were recruited in the first trimester at the Rheumatology and Reproduction (RhePro) interdisciplinary outpatient care unit of the Department of Internal Medicine III, Division for Rheumatology and the Department of Obstetrics and Gynecology of the Medical University of Vienna. Healthy pregnant women were enrolled at the birth registration between the 11th and 14th gestational weeks at the Department of Obstetrics and Gynecology of the Medical University of Vienna.

Women suffering from other autoimmune diseases besides SARD, such as inflammatory bowel diseases, multiple sclerosis, Graves’ disease, and malignancies during the pregnancy, were not included in this study. All patients were enrolled upon signing an informed patient consent within the time frame of 1 January 2022 and 30 July 2025. SLE was diagnosed according to the 2019 EULAR/ACR classification criteria (17), RA according to the 2010 EULAR/ACR classification criteria (18), spondyloarthritis according to the ASAS classification criteria (19), and psoriatic arthritis according to the CASPAR classification criteria (20). Disease activity during pregnancy was assessed using SLEDAI-2K in patients with SLE (21), CDAI in patients with RA (22), DAPSA in patients with psoriatic arthritis (23), and BASDAI in patients with spondyloarthritis (24). Active disease was defined as a SLEDAI-2K score >6, a CDAI score >10 (22), or a BASDAI score >4 (24), respectively. Disease activity was assessed once per trimester. If the predefined threshold was exceeded at any assessment, the disease was classified as active during pregnancy. Additionally, we took into account the increase in inflammatory parameters (CRP) and changes in medications (for example, newly introduced glucocorticoid administration or dose elevation) during pregnancy. Based on this information, disease activity of every affected woman was assessed individually. All of the rheumatologic patients were followed throughout the pregnancy by an interdisciplinary team consisting of a rheumatologist and gynecologist.

### 2.2 Sample preparation

Immediately after birth, fresh serum and EDTA blood of the UC vein (UCV) were collected. Additionally, tissue of the umbilical cord was fixed by 4.5% buffered formaldehyde (Sigma-Aldrich, St. Louis, MO, USA) after washing in PBS to remove blood. Umbilical cords were further used for isolation of human umbilical cord vein cells (HUVECs) and human umbilical cord smooth muscle cells (HUVSMCs). Serum was isolated by centrifugation, and aliquots were stored at −80 °C until further analysis. A small amount of fresh blood was used as plasma, and PBMCs were isolated by a Ficoll gradient centrifugation. PBMCs were frozen in fetal calf serum (FCS; Linaris, LaborChemie, Vienna, Austria) and 10% DMSO (Sigma-Aldrich, St. Louis, MO, USA) in liquid N_2_, and plasma was stored at −80 °C until further analysis.

### 2.3 Histology

Sections of paraffin-embedded UC (3 µM) were stained with H&E staining (Merck, Darmstadt, Germany) and Elastica van Gieson (Merck, Darmstadt, Germany) kits. Sections were embedded in Entellan (Sigma-Aldrich, St. Louis, MO, USA) and analyzed in a Nikon C2 confocal microscope (Nikon Instruments, the Netherlands).

### 2.4 Immunofluorescence

Five-micrometer sections of paraffin-embedded UC were used for immunofluorescence. Briefly, sections were deparaffinized at 60 °C for at least 2 h, demasked in KOS microwave (Milestone, Italy) at 95 °C and in citrate buffer, and washed with PBS/0.1% Tween. Tissue was permeabilized using 0.2% Triton X 100/PBS (Sigma-Aldrich, St. Louis, MO, USA) for 5 min RT, washed, and blocked with 10% goat serum solution in 1× PBS (Thermo Fisher Scientific, Vienna, Austria). Primary antibodies were diluted in blocking buffer [anti-human CD31 (polyclonal) and anti-human intercellular adhesion molecule-1 (ICAM-1) (monoclonal) from PTG and THP, Vienna, Austria]. Secondary antibodies used were Alexa 488-conjugated goat anti-mouse or anti-rabbit (Thermo Scientific, Vienna, Austria) depending on the first antibody. Nuclei were visible by incubation with Hoechst stain 33342 (Life Technologies, Thermo Scientific, Vienna, Austria) in TBS for 20 min. Sections were embedded in mounting buffer (ProLong Antifade, Life Technologies, Thermo Scientific, Vienna, Austria) and analyzed in a Nikon confocal C2 camera using the ND2 software (Nikon Instruments, the Netherlands).

### 2.5 Flow cytometry

Hundred microliters of fresh UCV EDTA-blood samples were incubated with anti-human monoclonal (CD14 PE-Cy7, CD16 PerCP-Cy5.5,CD45 APC-H7, and HLA-DR BV 421, all from BD Biosciences, San Diego, USA) antibodies. Monocytes were gated by forward and site scatter, singlets, and C45-positive cells, and HLA-DR-negative cells were analyzed for CD14 and CD16 expression in a BD FACSCanto II flow cytometer (BD Biosciences, San Diego, USA). The percentage of CD14 and CD16 or double-positive cells was calculated using the BD FACS DIVA and FlowJo software (BD Biosciences, San Diego, USA). Frozen leukocytes were carefully thawed in a water bath and immediately diluted in PBS and 1% FCS and centrifuged. Cells were resuspended in PBS with 1% FCS and stained with the following antibodies: CD45 APC-H7, CD19 V450, and CD4 FITC (BD biosciences, San Diego, USA), as well as CD25 PE and CD3 AF700 (Life Technologies, Thermo Fisher Scientific, Vienna, Austria).

### 2.6 Cytokine array

The Proteome Profiler Human XL Cytokine Array Kit (R&D Systems, Bio-Techne, Ireland) was used to evaluate the cytokine profile in the UCV serum of newborns. Sera were pooled for each group and diluted with a wash buffer (1:15) according to the manufacturer’s instructions. The signals were detected using the Vilber chemiluminescence imaging (Vilber, Germany) and detected and quantified using the Vilber software (Bio One, Vilber, Germany) in pixels as arbitrary units (AU).

### 2.7 Enzyme-linked immunosorbent assay

Enzyme-linked immunosorbent assay (ELISA) specific for TNF-α, platelet-derived growth factor AA (PDGF-AA), IL-8, and IL-4 was performed according to the manufacturer’s instructions (all from R&D Systems, Bio-Techne, Ireland). Briefly, undiluted sera of patients were loaded onto coated microtiter plates in duplets and standards (NUNC, Thermo Scientific, Vienna, Austria). Biotinylated secondary antibodies were incubated and signals were detected with streptavidin-conjugated substrate and measured at 450 nm on a Tecan Spark ELISA Reader (Tecan, Switzerland) and quantified with standards.

### 2.8 Cell culture

HUVECs and HUVSMCs were isolated as follows: UC veins were cannulated and washed with PBS. UCVs were filled with collagenase (147.5 U/mL PBS, 3–5 mL per UC), closed, and incubated for 20 min in an incubator at 37 °C. Cells were flushed with RPMI media + 10% FCS and centrifuged. Cells were resuspended in EC media + supplements according to the manufacturer’s instructions (Provitro and THP, Vienna, Austria) and incubated in T25 bovine collagen (0.2% in PBS) (Sigma-Aldrich, St. Louis, MO, USA) (coated flasks until confluency) and frozen for further analysis. HUVSMCs were isolated from EC-free UCV, minced, and outgrown in gelatin-coated 6-well plates until confluency. HUVSMCs were further transferred to 25T flasks and grown until confluency in SMC media (Thermo Scientific, Vienna, Austria) and frozen for further analysis.

### 2.9 XTT proliferation assay

HUVECs and HUVSMCs were thawed and cultured until confluency. Cells were seeded in 96-well plates and grown for 24 h on 0.2% gelatin-coated plates and cultured in specific media. Cells were incubated with either recombinant (r)IL-8 (BioLegend, Vienna, Austria) at different concentrations (100 pg, 1 ng, 500 pg, and 100 pg, respectively, diluted in respective media) or 10% plasma from UCV. After 16 h of incubation, dissolved XTT sodium salt powder (Thermo Scientific, Vienna, Austria) and phenazine methosulfate (PMS) (at 5 µM) were added to the medium. The plates were incubated for an additional 4 h, and the optical density was measured at 450 nm (reference wavelength −595 nm) using a Tecan reader (Tecan, Morgan Hill, CA, USA). For quantification, the background levels of media without cultured cells were subtracted.

### 2.10 ROS assay

The DCFDA/H2DCFDA-cellular ROS (reactive oxygen species) (Abcam, Waltham, MA, USA) was used according to the manufacturer’s instructions. Briefly, 1 × 10^5^ HUVEC cells were grown ON per well in 0.2% gelatin-coated 96-well plates from healthy donors and washed with buffer 100 µL per well. Cells were incubated with DCFD solution for 45 min at 37°C (in the dark). Cells were washed and incubated with different concentrations of UCV plasma from healthy, IJD, and CTD offspring for 4 h. As positive control, TBMP and 0.3% H_2_O_2_ were used. Experiments were done twice in doublets and analyzed using fluorescence spectroscopy with excitation/emission at 485/535 nm in a Tecan reader (Tecan, Morgan Hill, CA, USA).

### 2.11 Statistical methods

Maternal sociodemographic, clinical, and obstetric characteristics were summarized for the overall cohort and stratified into three groups: healthy controls, IJD group, and CTD group. Continuous variables such as maternal age, prepregnancy BMI, gestational age at delivery in weeks, and neonatal weight are presented as mean and standard deviation (SD). Categorical variables including parity, mode of conception, nicotine and alcohol consumption, disease activity, medication use during pregnancy, and mode of delivery are reported as counts and percentages.

Between-group comparisons for continuous variables were performed using ANOVA and for categorical variables chi-square or Fisher’s exact, as appropriate. Differences between healthy controls, the IJD group, and the CTD groups were explored using two-sided tests with a significance level of *p* ≤ 0.05 and a Bonferroni correction, when needed. Statistical analyses were performed using Microsoft^®^ Excel. For ELISA and flow cytometry data, statistical analyses were performed using ANOVA and *post hoc* test or Kruskal–Wallis test in GraphPad Prism (GraphPad Software).

Exploratory medication–cytokine association analyses were conducted using individual IL-8 measurements from patients for whom both cytokine and medication data were available. Within the IJD group, IL-8 concentrations were compared between patients receiving and not receiving TNF-α inhibitors. Within the CTD group, IL-8 concentrations were compared between patients receiving and not receiving hydroxychloroquine. Because IL-8 values were not normally distributed and the medication subgroups were small, results are presented as medians and interquartile ranges (IQRs), and between-group comparisons were performed using the Wilcoxon rank-sum tests. IL-8 concentrations were additionally explored according to disease activity in the IJD group. Analyses of medication categories represented by only a small number of patients were restricted to descriptive reporting. These exploratory analyses were performed using R (version 4.6.0) (R Foundation for Statistical Computing, Vienna, Austria), and two-sided *p*-values below 0.05 were considered statistically significant.

## 3 Results

### 3.1 Descriptive characteristics of the study population

Within the time frame of our study, 103 patients met the inclusion criteria. Sixteen women were excluded due to missing samples or missing core data.

Therefore, after applying the exclusion criteria, 87 women remained (**Supplementary Figure 1**, flowchart). In the group with IJD, 25 patients were available (10 RA, 4 JIA, 6 axSpA, 3 peripheral SpA, and 2 PsA); in the group with CTD, 23 patients were available [14 SLE (1 suffered additionally from APLAS and 1 additionally from SS), 6 SS, 2 systemic sclerosis, and 1 mixed connective tissue disorder with APLAS); and in the HC group, 39 women were available (**Table 1**).

**Table 1:** Characteristics of the study population.

Variables	IJD	CTD	HC	*p*
***N***	25	23	39	
**BMI (kg/m^2^) (SD)**	23.18 ( ± 3.37)	26.15 ( ± 7.75)	24.33 ( ± 6.02)	0.225
**Maternal age at delivery (years) (SD)**	34.00 ( ± 4.74)	33.5 ( ± 4.38)	32.05 ( ± 5.22)	0.254
**Smoking, *n* (%)**	0 (0.00)	2 (8.70)	2 (5.13)	0.316
**Alcohol, *n* (%)**	2 (8.00)	0 (0.00)	2 (5.13)	0.426
**Parity, *n* (%)**				
Primiparae, *n* (%)	9 (36.00)	9 (39.13)	8 (20.51)	0.178
Multiparae, *n* (%)	16 (64.00)	13 (56.52)	31 (79.49)	0.222
**Disease flare during pregnancy, *n* (%)**	8 (32.00)	0 (0.00)	n.a.	0.002*
**Immunomodulatory** **drug use, *n* (%)**	21 (84.00)	17 (73.91)	0 (0.00)	0.401*
Hydroxychloroquine/ chloroquine, *n* (%)	1 (4.00)	17 (73.91)	0 (0.00)	
Glucocorticoids, *n* (%)	7 (28.00)	3 (13.04)	0 (0.00)	
Azathioprine, *n* (%)	1 (4.00)	3 (13.04)	0 (0.00)	
Sulfasalazine, *n* (%)	3 (12.00)	0 (0.00)	0 (0.00)	
Biologics, *n* (%)	13 (52.00)	1 (4.35)	0 (0.00)	
**Mode of delivery, *n* (%)**				
C-section, *n* (%)	15 (60.00)	9 (39.13)	20 (51.28)	0.433
Operative vaginal, *n* (%)	3 (12.00)	4 (17.39)	3 (7.69)	0.498
Spontaneous vaginal, *n* (%)	7 (28.00)	9 (39.13)	17 (43.59)	0.486
**Pregnancy outcome**				
Neonatal weight (g) (SD)	3,150.08 ( ± 562.05)	3,319.36 ( ± 564.36)	3,347.2 ( ± 446.03)	0.303
Gestational age (weeks) (SD)	38.64 (± 1.85)	39.32 (± 1.46)	39.75 ( ± 1.30)	0.019**
Preeclampsia, *n* (%)	0 (0.00)	2 (8.70)	0 (0.00)	0.056
Preterm birth, *n* (%)	3 (12.00)	1 (4.35)	1 (2.56)	0.267
GDM, *n* (%)	2 (8.00)	4 (17.39)	6 (15.38)	0.611

*p*-values ≤ 0.05 are considered statistically significant and are displayed in bold.

IJD, inflammatory joint disease; CTD, connective tissue disorder; HC, healthy control; GDM, gestational diabetes mellitus.

*p-values calculated between the IJD and CTD groups only significant differences in bold.

**Bonferroni *post hoc* test: HC *vs*. IJD, *p* = 0.019.

No statistical difference was seen between the IJD, CTD, and HC groups based on maternal descriptive characteristics such as prepregnancy BMI, maternal age, nicotine consumption, number of spontaneous conceptions, mode of delivery, and number of primiparae. Disease flare during pregnancy was significantly more frequent in the IJD group compared with the CTD group (*p* = 0.002). The most common immunomodulatory medication used during pregnancy was hydroxychloroquine (HCQ), administered in 73.91% of patients with CTD. This was followed by azathioprine (AZA) and glucocorticoids, each used in 13.04% of patients. In IJD patients, 52.00% received biologics during pregnancy, followed by glucocorticoids (28.00%) and sulfasalazine (12.00%).

Rates of preeclampsia (PE) and gestational diabetes mellitus (GDM) did not show any significant differences between the IJD, CTD, and HC groups. Furthermore, no difference was detected between the groups in neonatal weight at delivery. A statistically significant difference in gestational age at birth was seen between the control and IJD groups (mean: 39.75 *vs*. 38.64 weeks, HC *vs*. IJD, *p* = 0.019).

The exact diagnosis, disease activity, and medication use during pregnancy are shown in detail in **Supplementary Table 1**.

### 3.2 *In situ* ICAM-1 expression in umbilical cord tissue as a sign of inflammation

**Figure 1:**
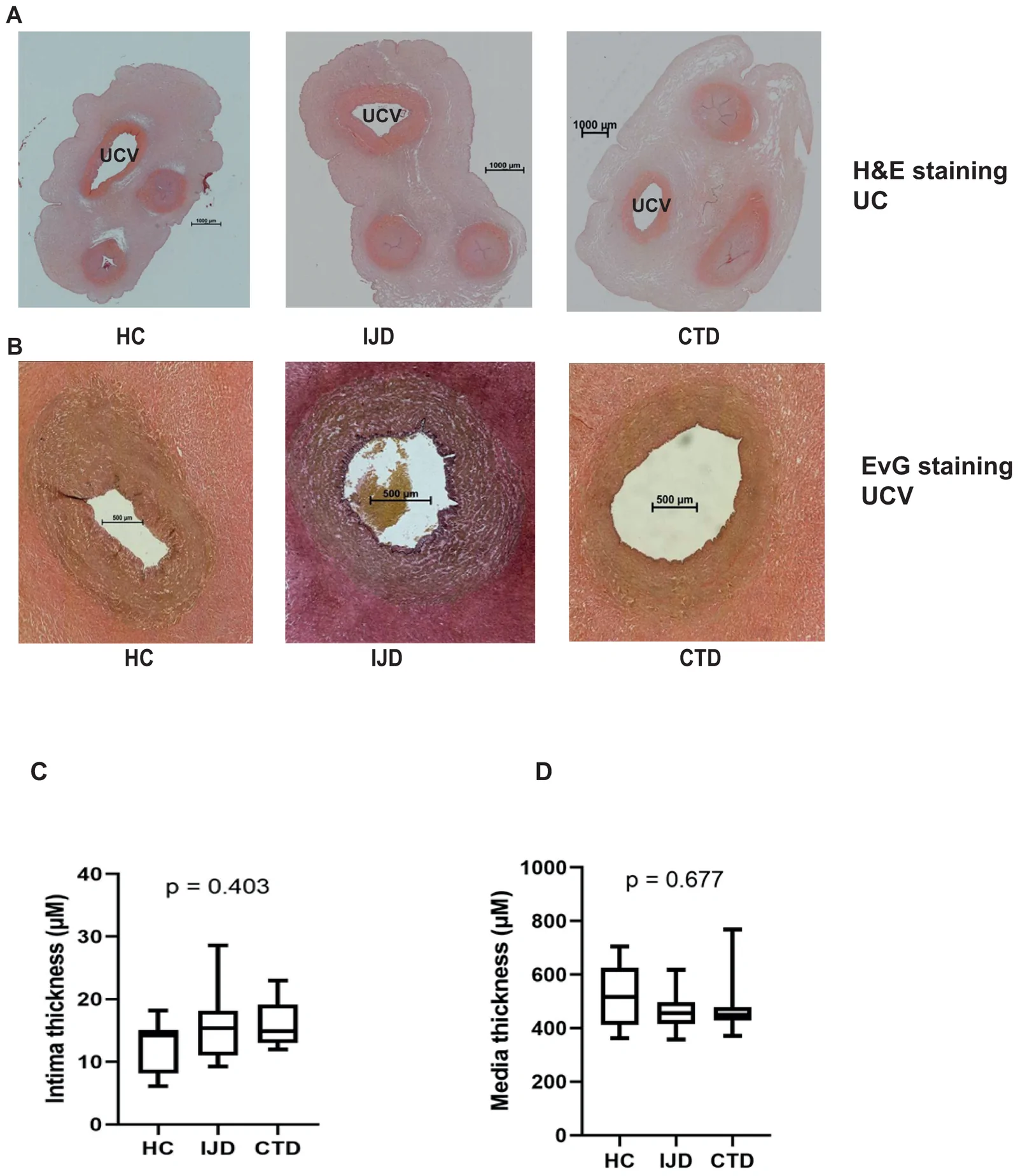
Differences in histology of umbilical cord (UC) and veins (UCV). **(A)** H&E staining and **(B)** Elastica van Gieson (EvG) staining of umbilical cord sections of one representative sample of each group. HC, healthy control; IJD, inflammatory joint disease; CTD, connective tissue disorders. **(C)** Quantification of intima thickness and **(D)** media thickness of umbilical cord veins (*n* = 7 of each group). Data are demonstrated in box blots (median and SD).

To analyze the immediate effect of the underlying autoimmune disease of the mothers on the vasculature of the offspring, we used the UCV as a model for the neonatal vasculature. We performed histological staining (H&E) of the UCV tissue of different groups (*n* = 7 each group) (**Figure 1A**). In stained tissue, no leukocyte infiltrations were visible, as described, e.g., in infections. To investigate the effect of maternal disease on the vascular smooth muscle cells (VSMC) we performed Elastica van Gieson (**Figure 1B**) staining. No significant differences in media thickness (**Figure 1C**) were detectable.

**Figure 2:**
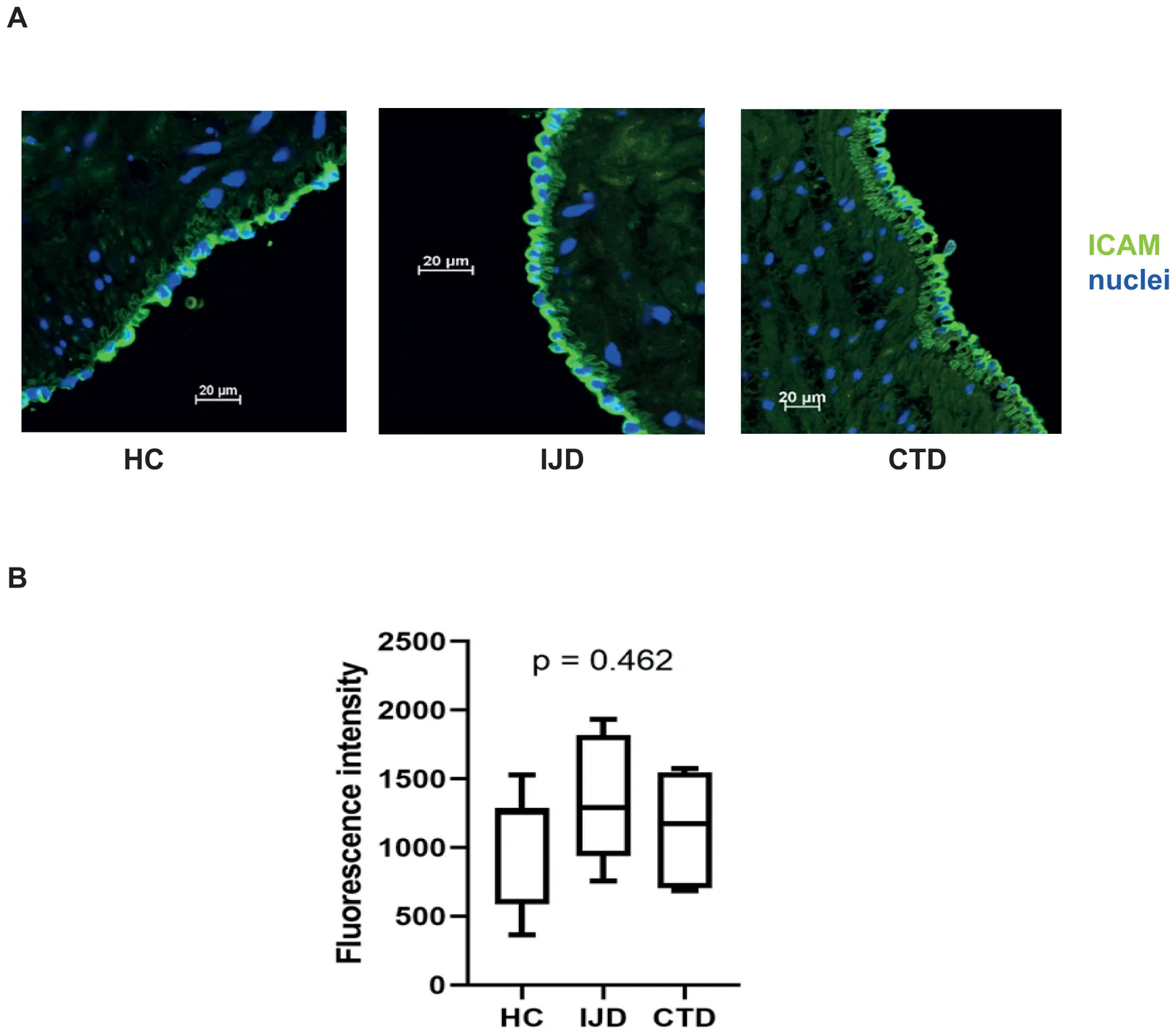
ICAM expression in umbilical cord veins. **(A)** ICAM expression of endothelium of umbilical cord veins in immunofluorescence as one representative picture of each group. **(B)** Quantification of the fluorescence intensity of ICAM expression (*n* = 8) and quantified by ANOVA. Data are demonstrated in box blots.

Additionally, we analyzed the expression levels of adhesion molecules in the endothelium, known to be upregulated under inflammatory conditions. We found a tendential increase of ICAM-1 expression in immunofluorescence (*p* = 0.462), in the endothelium of UCV and in the tissue of UC of IJD patients, indicating a potential increase of inflammation in response to inflammatory cytokines within this group of patients (**Figures 2A, B**). The endothelium was confirmed by CD31 staining in UCV (data not shown).

### 3.3 Immune cell levels in venous blood of umbilical cords

**Figure 3:**
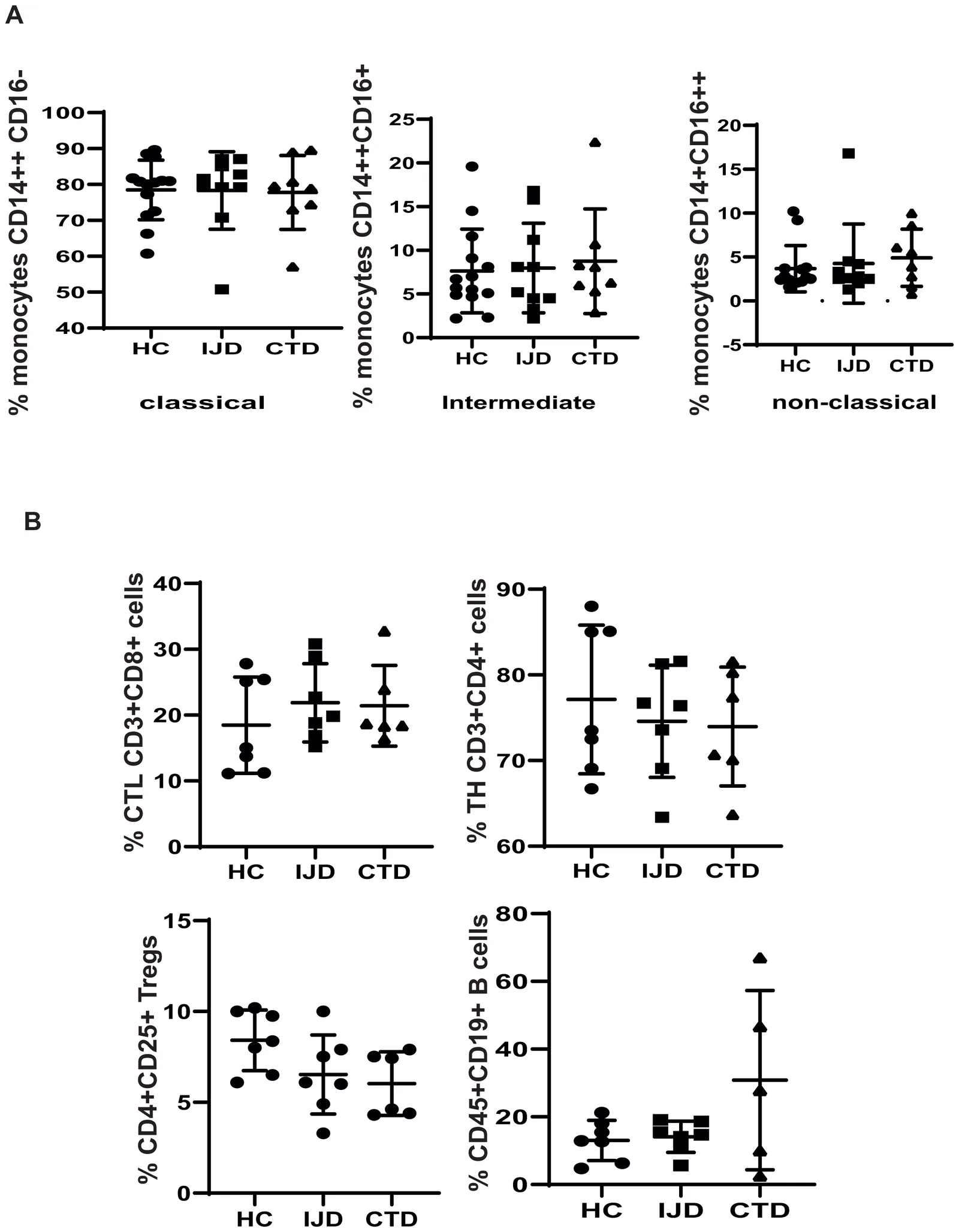
Monocyte and other immune cells of venous cord blood quantified by flow cytometry. **(A)** monocyte subsets and **(B)** cytotoxic lymphocytes (CTL), T helper (TH) cells, regulatory T cells (Tregs), and B cells are demonstrated by scatter blots. Differences between the groups were not significant.

Upon inflammation, several immune cells change their profile. In our study, we compared classical, intermediate, and non-classical monocytes in flow cytometry in fresh venous cord blood of neonates of healthy patients and patients with IJD and CTD. In all subsets of monocytes, no difference between the groups was detectable (**Figure 3A**). Furthermore, we compared frozen leucocytes for markers specific for regulatory T cells (Tregs), T helper cells, cytotoxic T lymphocytes, and B cells in different groups of VUCB (**Figure 3B**). No significant difference in all lymphocyte subsets was detectable, although a tendential reduction of Tregs and an increase of CTL cells were found in IJD and CTD blood. Interestingly, an increase of B cells was detectable in the CTD group of lymphocytes of UC blood.

### 3.4 Cytokine profile in the UCV sera of neonates

**Figure 4:**
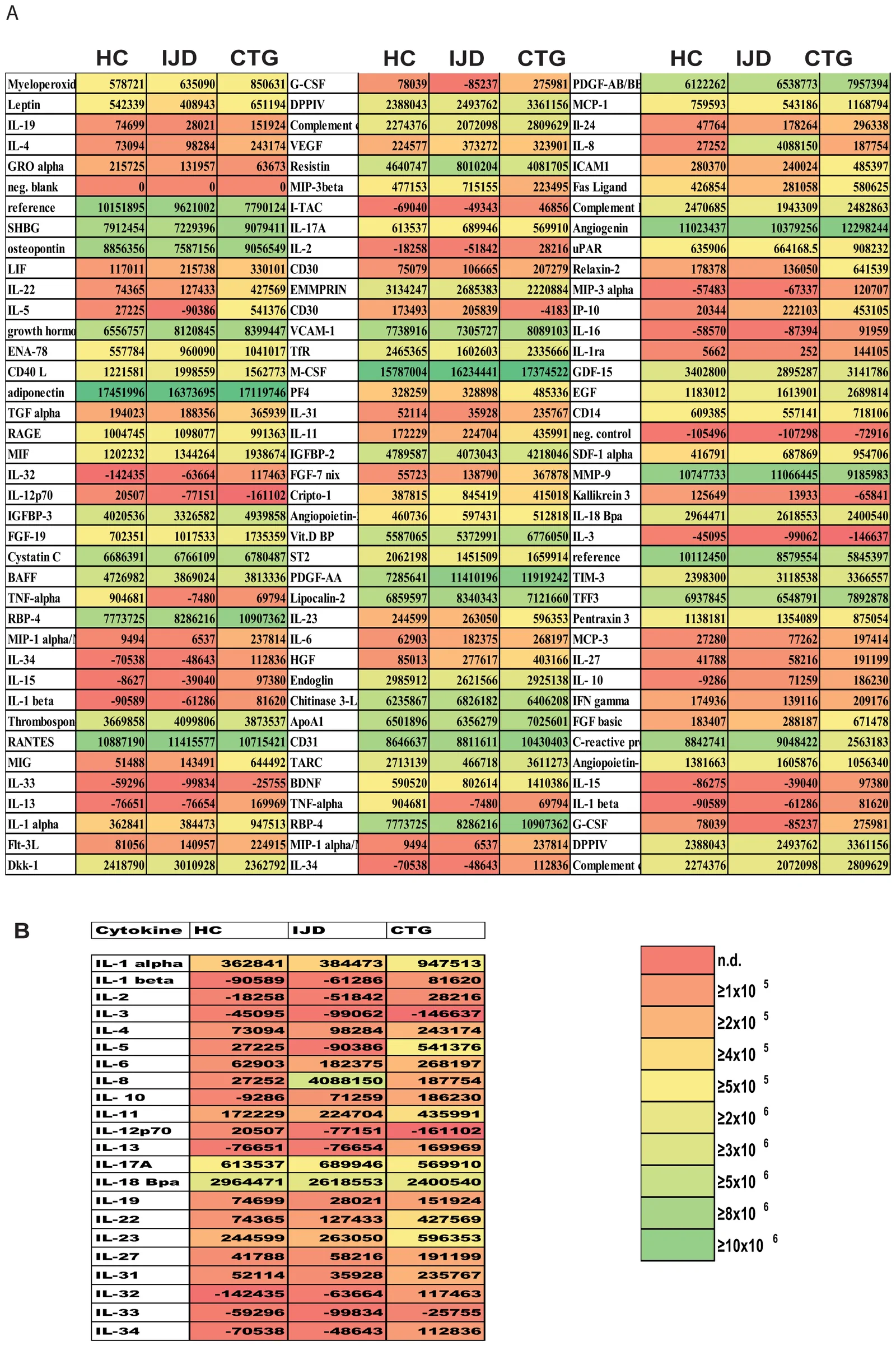
Cytokine/other mediator levels of sera of UCV blood were evaluated. Pooled sera of UCV blood of different groups of patients were tested in an array (*n* = 7, each group). HC, healthy controls; IJD, inflammatory joint diseases; CTD, connective tissue disease. **(A)** Levels of all cytokines/soluble mediators are demonstrated in colors depending on the level of arbitrary units. **(B)** Interleukins were demonstrated separately in arbitrary units. Differences are shown as a relationship of values to healthy controls.

**Figure 5:**
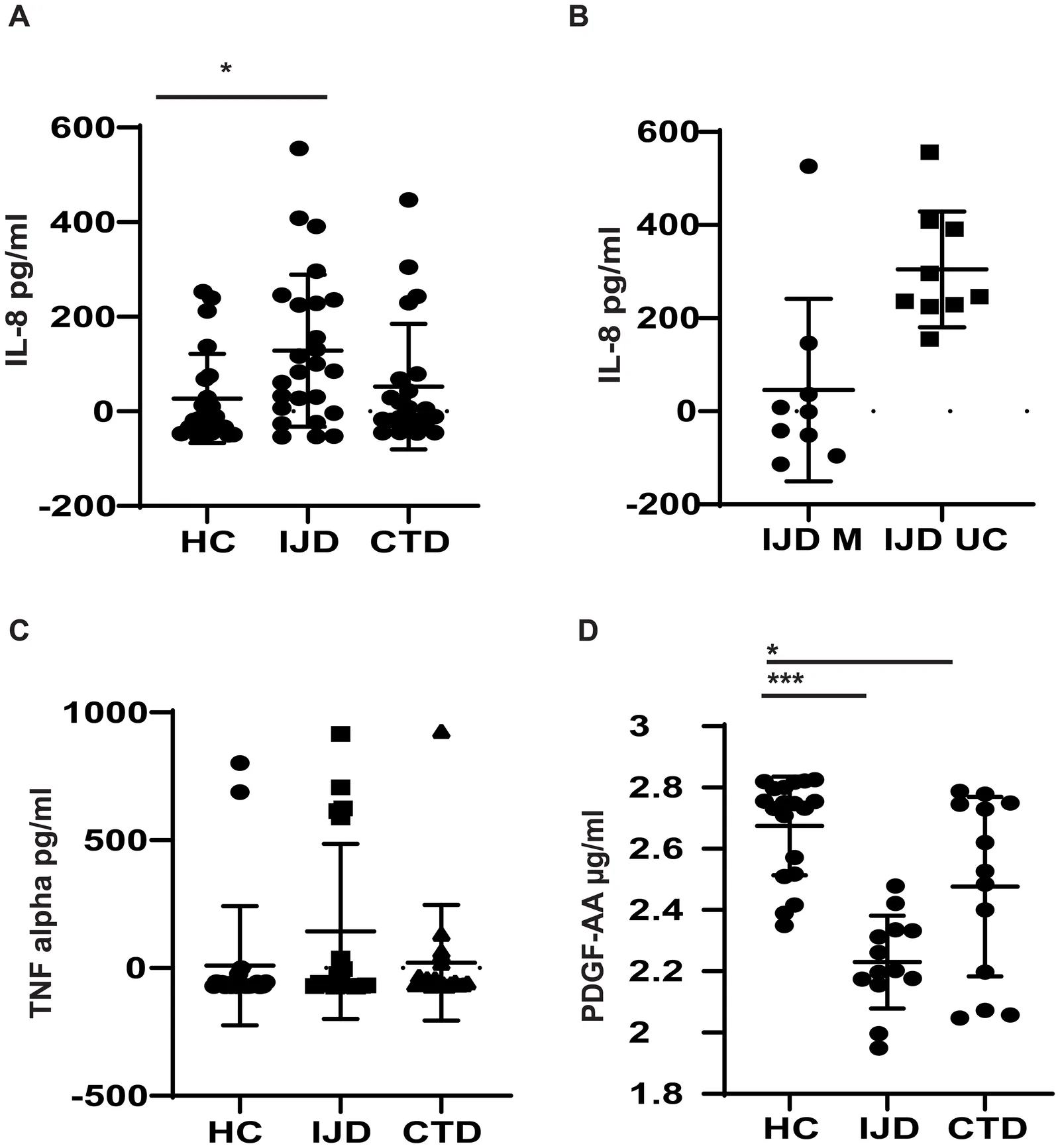
Cytokine levels in the sera of umbilical cord vein blood quantified by ELISA. PDGF-AA levels are significantly reduced and IL-8 levels are significantly elevated in the sera of IJD newborns. **(A)** TNF-α levels; **(B)** PDGF-AA levels (***HC *vs*. IJD p = 0.0001, *HC *vs*. CTD p = 0.02). **(C)** IL-8 levels in the sera of UCV blood (*HC *vs*. IJD p = 0.0129). **(D)** IL-8 levels in the sera of IJD mothers compared to UCV sera at birth. Data are demonstrated in scatter blots.

To verify if soluble factors and mediators, such as cytokines, show differences in the IJD and CTD groups *vs*. HC in UCV, we performed a cytokine array with pooled sera of each group of UC samples in a semiquantitative manner (**Figure 4A**; **Supplementary Figure 2**). Several mediators were highly expressed without dramatic differences. The level of interleukins in serum was relatively low in all groups (**Figure 4B**). Differences between the groups of pooled sera (each *n* = 7) did not differ dramatically, although an increase of IL-8 in the IJD group with a 150-fold level compared with the HC group (**Figure 4B**) was detectable. IL-8 ELISAs confirmed our observation, showing significantly higher IL-8 levels in the sera of UCV in the IJD group compared with the HC group (**Figure 5C**). To determine whether IL-8 might be of maternal origin, we compared the high-level IL-8 sera of the UCV in the IJD group with the sera of the corresponding mothers (**Figure 5D**). Interestingly, the high levels of IL-8 detected in the UCV were not observed in maternal serum.

Individual IL-8 and medication data were available for exploratory analyses in 22 patients with IJD and 21 patients with CTD. Within the IJD group, IL-8 concentrations did not differ significantly between patients receiving and not receiving TNF-α inhibitors (median 32, IQR −24 to 225, *n* = 13 vs. median 131, IQR 61 to 236, *n* = 9; *p* = 0.423). Within the CTD group, IL-8 concentrations did not differ significantly between patients receiving and not receiving hydroxychloroquine (median −13, IQR −33.5 to 61, *n* = 15 vs. median 18.5, IQR 5.75 to 58.25, *n* = 6; *p* = 0.311). In the IJD group, IL-8 concentrations also did not differ significantly between patients with active and inactive disease (median 108, IQR −2.75 to 255, *n* = 6 vs. median 93, IQR 22.75 to 228, *n* = 16; *p* = 0.796). Because the remaining medication categories comprised only a small numbers of patients, no further medication-specific inferential comparisons were performed.

These analyses did not provide evidence of a statistically significant association between the principal medication categories and neonatal IL-8 concentrations.

Very low TNF-α levels were measured in the UCV, and no difference between the groups was detected (**Figure 5A**). Furthermore, IL-4 levels were very low in all groups and did not show any significant difference between the groups (**Supplementary Figure 3**).

A significant reduction of PDGF-AA in the sera of IJD and CTD neonates compared to the newborns of healthy controls was detectable determined by ELISA (HC *vs*. IJD *p* = 0.0001, HC *vs*. CTD *p* = 0.02) (**Figure 5B**).

### 3.5 Plasma of IJD offspring triggers reduced proliferation of HUVSMCs

**Figure 6:**
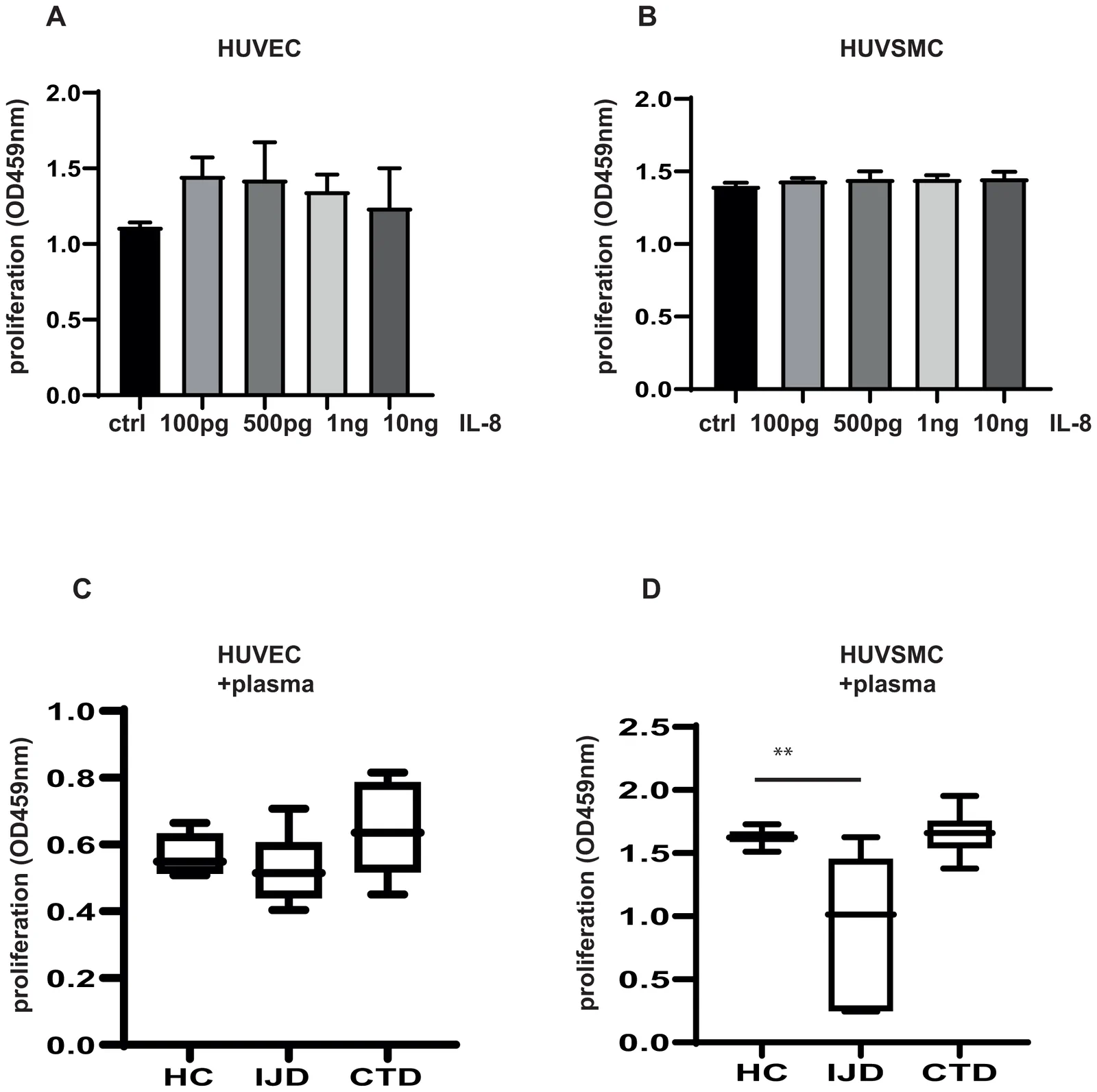
Proliferation of HUVECS and HUVSMCS of healthy control with **(A)** increasing amounts of rIL-8 added to HUVECS and **(B)** HUVSMC incubated with increasing amounts of rIL-8. **(C)** HUVEC of HC incubated with plasma of UC of healthy individuals (HC), plasma of IJD UC blood, and plasma of CTD UC blood. **(D)** HUSMC of HC incubated with plasma of UC of healthy individuals (HC), plasma of IJD UC blood, and plasma of CTD UC blood (p = 0.0027). Data of two independent experiments.

Oxidative stress and inflammation were directly involved in tissue damage. Therefore, we tested the amount of ROS in HUVECs after exposure to plasma of the UCV of newborns of IJD or CTD mothers, where no difference was visible (data not shown). To determine the effect of IL-8 on the proliferative potential of endothelial cells and SMCs of the umbilical cord vein in healthy controls, we performed an XTT assay. Different concentrations of recombinant IL-8 had a low effect on HUVECs but no effect on the proliferation of HUVSMCs (**Figures 6A, B**). Furthermore, after the incubation of UCV plasma of IJD and CTD and HC with HUVEC of HC, we did not detect any effect on the proliferation in HUVECs (**Figure 6C**). Interestingly, treatment with UCV plasma of IJD neonates induced a significantly reduced proliferation rate in HUVSMCs of HC, compared with UCV plasma of healthy controls (*p* = 0.027) (**Figure 6D**). These data suggest that elevated IL-8 levels in the sera of IJD umbilical cord vein blood do not affect the proliferative capacity or survival of HUVECs and HUSMCs.

## 4 Discussion

SARDs in pregnant women are associated with a higher risk of pregnancy complications, such as preeclampsia, fetal growth restriction, and preterm birth. These complications are rooted in placental pathologies associated with pathological placentation at early pregnancy or in the inflammation and malfunction of placental vasculature at later times throughout the pregnancy. The elevated risk of these complications can be associated with maternal chronic inflammation, autoantibody production, and high disease activity during pregnancy (12, 25). Additionally, impaired endothelial function was shown in children suffering from rheumatic diseases (26). No studies investigated the effect of systemic autoimmune rheumatic diseases on the newborn vasculature directly after birth. As a model of fetal vasculature, we used UC tissue, the main vessel of the fetus.

We found no signs of inflammatory infiltrations in the UC tissue; however, there was a tendential increase of ICAM-1 expression in the UCV of the IJD group. This might indicate disbalanced signaling in the endothelium in the UCV.

Leukocyte subpopulations differ in patients with IJD and CTD compared with healthy individuals: non-classical monocytes are known to be elevated in peripheral blood of patients with IJD and intermediate monocyte subsets in patients with SLE (27–29). In UCV blood, no differences in monocyte subtypes in the IJD and CTD groups were detected in our study, as described by others (30). An increased number of B cells was detected in UCV blood from the offspring of patients with CTD compared with healthy controls. This increase did not correlate with underlying disease activity. However, the number of samples was relatively small, and both groups showed heterogeneity in specific underlying disease types. Therefore, these results should be interpreted with caution. Our finding, however, is comparable to another study (31).

Increased concentrations of pro-inflammatory cytokines such as TNF-α, IL-6, IL-1beta, and IL-8 were detected in the maternal–fetal interface such as in the myometrium, placenta, and umbilical cord venous blood in laboring compared to non-laboring women (32). Therefore, increased cytokine levels might indicate the physiological state necessary for labor onset (33). However, dysregulated cytokine levels due to the rheumatic disease itself can lead to perinatal complications including preterm rupture of the membranes, preterm birth, preeclampsia, and impaired neonatal and impaired childhood neurodevelopment (34). Several maternal pathological conditions may influence offspring outcomes through remodeling of the pregnancy immune microenvironment. *In vitro* and *in vivo* studies suggest that infections (for example, hepatitis B infection or HIV infection) alter trophoblast apoptosis pathways and multiple inflammatory pathways, which in turn elevate the risk of obstetric and neonatal pathological conditions (35, 36).

The origin of increased cytokines in newborns remains unclear, and data suggest either a secretion in the UCV, an increased inflammatory response in the fetus around delivery, or a transplacental passage from the mother (37). IL-8 levels in the VUCB from neonates of healthy mothers are typically low, regardless of mode of delivery (38). In contrast, VUCB from neonates born to mothers who suffer from rheumatic diseases (including SLE, RA, SS, and systemic sclerosis) frequently shows markedly elevated IL-8 levels, often as part of a broader hypercytokinemia. The elevation was not mirrored in maternal blood and is believed to originate from fetal immune tissues rather than maternal or placental sources (39). Nevertheless, the transplacental passage of cytokines is still controversial. In our study, the high levels of IL-8 detected in the UCV were not observed in the corresponding maternal serum, indicating *de novo* production of IL-8 within the UC vasculature. Supporting this finding, a previous study demonstrated the absence of an active transport of IL-8 through the feto-maternal interface (40).

According to the EPIC-Norfolk study of healthy individuals, elevated baseline IL-8 concentrations predict future coronary artery disease (CAD). They found that individuals in the highest IL-8 quartile had a 77% increased odds of future CAD (41). Among patients with established coronary disease, IL-8 was the only cytokine among 10 measured that independently predicted cardiovascular events during a 7-year follow-up (42). This association renders further investigations concerning the relevance of SARD in future CVD risk in the offspring.

IL-8, furthermore, induces survival, proliferation, and angiogenesis in endothelial cells (43). We did not detect an increased proliferation potential in HUVECS and VSMCs of healthy UCVs in response to increased IL-8 signal. On the contrary, proliferation rates of VSMCs of healthy UCV incubated with plasma of IJD UC plasma showed a significant reduction. This might be, but not necessarily, a direct effect of the strongly reduced PDGF-AA levels in the IJD newborn group. PDGF-AA is a mitogen and chemoattractant for mesenchymal cells, acting mainly via PDGFRα to regulate proliferation and migration mainly in vascular SMC (44).

### 4.1 Immunomodulatory treatment of mothers and impact on cytokine levels

In our study population, 84.4% of all patients with rheumatic diseases received immunomodulatory therapy during pregnancy. Immunomodulatory medication, such as HCQ (45), glucocorticoids (46), and biologics (47) commonly used to treat rheumatic diseases, reduce the levels of pro-inflammatory cytokines. Studies failed to show such a clear decrease during AZA therapy (48). An increase of NF-kappa B was seen in placentas treated with AZA. Therefore, cytokine levels measured in our study might be modulated by these treatments.

The low TNF-α levels observed in our data are concordant with other studies, where TNF-α levels in umbilical cord blood were low, unrelated to labor status, and comparable between neonates born to healthy mothers and those born to mothers with rheumatoid arthritis or other autoimmune diseases, unless there is active disease, infection, or fetal distress (39, 48–50). Likewise, IL-6 levels were very low in all groups of UC serum of neonates in our study. TNF-α inhibitors directly neutralize circulating TNF-α and block its receptor-mediated effects, leading to decreased systemic inflammation and improved disease control. Thirteen out of 25 IJD patients in our study population were treated with TNF-α blockers and 19 out of 25 patients were in remission. Some TNF-α blockers cross the rife placenta, but the fact that there was no difference detected between the IJD and CTD groups suggests that TNF-α is generally low in newborns at birth and this observation is possibly not a medication effect.

IL-4 concentration was not different between the control and the index groups in our study. IL-4 concentration is controversially discussed in studies of patients with rheumatic diseases. Some studies detected higher IL-4 levels in the serum of patients with rheumatic diseases, and IL-4 deficiency might be advantageous in RA. In pregnancy, *in vitro* studies associated preeclampsia with a defective IL-4/IL-10 signaling pathway.

Our findings reassuringly suggest that only limited maternal inflammatory response is transferred to the fetus at birth, which indicates the importance of successful disease management during pregnancy. Nevertheless, the extensive *de novo* synthesis of IL-8 observed in our study might have an impact on the vasculature of offspring of IJD mothers. After exploring possible confounding factors, we found no evidence of a statistically significant association between the principal medication categories and neonatal IL-8 concentrations. Given the small and unequal subgroup sizes, the analyses should be regarded as exploratory and cannot exclude medication-related effects.

Our observed finding might represent a short-term alternation of the local immunological status; however, as IL-8 is a potent predictor of cardiovascular disease in adults, further studies are needed to confirm that this elevation is only a short-time effect at birth, without long-lasting influence on the vasculature of the offspring. Long-term prospective follow-up of children born to mothers with SARD is still much needed concerning the impact of cytokine imbalances during pregnancy and after birth.

### 4.2 Limitations of the study

The relatively small sample size limits the implications on the various disease subtypes and therapy modalities on inflammatory markers of the newborn. Our classification into IJD and CTD groups was based primarily on their predominant clinical manifestations and established pathophysiological characteristics. Given the rarity of these diseases in pregnancy, disease-specific subgroup analyses were not sufficiently powered in the present cohort. Therefore, our findings should be interpreted as exploratory observations regarding broader categories of autoimmune rheumatic disease rather than as evidence for effects attributable to individual diagnoses. Moreover, our pregnant patient cohort represents well-controlled patients with no serious maternal or fetal complications and no major placental pathologies. For this reason, we have to limit our findings and conclusion to SARD patients of similar course of disease and course of pregnancy.

Given these complexities, further studies with larger cohorts are essential to better understand how different autoimmune diseases and immunomodulatory therapies impact maternal–fetal immune crosstalk.

## Data Availability

The raw data supporting the conclusions of this article will be made available by the authors, without undue reservation.

## Supplementary Material

Supplementary Figure 1 Flowchart of the inclusion and the exclusion criteria.

Supplementary Figure 2 Raw data of chemi-lumeniscence of the cytokine arrays as indicated.

Supplementary Figure 3 IL-4 levels in sera of umbilical cord vein blood quantified by ELISA.
