# Supplementary material for: Increased interleukin-8 levels in cord blood serum of newborn—possible effect of cytokine dysregulation in pregnancies complicated by rheumatic disease on newborn vasculature
Source: Front Immunol. 2026 Sep 8;17:1931512. doi: 10.3389/fimmu.2026.1931512 (PMC13593472; doi:10.3389/fimmu.2026.1931512)
Supplement: Supplementary file 1 [file DataSheet1.pdf]

Supplementary Table 1: Characteristics of the index group: Exact diagnosis, disease activity, and medication use

Variable	IJD	CTD
**Disease activity***	25	23
Active, n (%)	6 (24.0)	2 (8.70)
Not active, n (%)	19 (76.0)	21 (91.30)
**Immunomodulatory Medication**	21	17
HCQ, n (%)	1 (4.0)	17 (73.91)
Azathioprine, n (%)	1 (4.0)	3 (13.04)
Sulfasalazine, n (%)	3 (12.0)	0 (0.0)
TNF-a Inhibitors, n (%)	13 (52.0)	1 (4.35)
Glucocorticoids, n (%)	7 (28.0)	3 (13.04)

* Disease-activity during pregnancy was measured through SLEDAI-2K activity score in SLE patients, CDAI in RA, BASDAI in spondyloarthritis and DAPSA in psoriatic arthritis. An active disease was defined as a SLEDAI-2K value over 6, as a CDAI score over 10.0, a BASDAI score over 4, and DAPSA over 4. Disease activity was assessed once per trimester during pregnancy. If the disease activity score exceeded the predefined threshold at any assessment, the disease was classified as active during pregnancy.

	IJD		CTD
**Diagnoses***	25		23
**JIA, n (%)**	4 (16.0)	**SLE, n (%)**	14 (60.87)
**RA, n (%)**	10 (40.0)	**Sjögrens Disease, n (%)**	7 (30.43)
**Spondyloarthritis, n (%)**	11 (44.0)	**APLAS, n (%)**	2 (8.70)
axSpA, n (%)	6 (24.0)	**MCTD, n (%)**	1 (4.35)
pSpA, n (%)	3 (12.0)	**SSc, n (%)**	2 (8.70)
PsA, n (%)	2 (8.0)		

APLAS: antiphospholipid antibodies syndrome, axSpA: axial spondyloarthritis, CTD: connective tissue diseases, IJD: inflammatory joint diseases, JIA: juvenile idiopathic arthritis, MCTD: mixed connective tissue disease, PsA: psoriatic arthritis, pSpA: peripheral spondyloarthritis, RA: rheumatoid arthritis, SLE: systemic lupus erythematosus, SSc: systemic sclerosis

*SLE was diagnosed per 2019 EULAR/ACR criteria, RA patients via EULAR 2010 criteria, spondyloarthritis patients using ASAS criteria, psoriatic arthritis patients using CASPAR classification.

Abbreviations: BASDAI: Bath Ankylosing Spondylitis Disease Activity Index , CDAI:Clinical Disease Activity Index, DAPSA: Disease Activity in Psoriatic Arthritis, SLEDAI- 2K: Systemic Lupus Erythematosus Disease Activity Index
